# 

**Published:** 1901-07-20

**Authors:** 


### NOTICE TO CORRESPONDENTS.

All MSS., Letters, Books for Review, and other matters intended for the Editor should be addressed The Editor, "The Hospital," The Hospital Building, 28 & 29 Southampton Street, Strand, W.C.

The Editor cannot undertake to return rejected MS., even when accompanied by stamped directed envelope.

**Notice to Advertisers and Subscribers.**

All Advertisements, Orders for Copies of The Hospital, and Business Communications should be addressed to **THE MANAGER (Not to the Editor),** The Hospital, 28 & 29 Southampton Street, Strand, London, W.C.

The Cost of Subscription, post free, is as follows:—Per week, 2½d.; per quarter, 2s. 8d.; per half-year, 5s. 3d.; per annum, 10s. 6d. Foreign:—Per annum, 15s. 2d., payable, in all cases, in advance.

**NOTICE.—Vol. XXIX. of "The Hospital" (October 1900, to March, 1901), in handsome cloth binding is now on sale at the Office of this Journal, price, 7s. 6d. Cases for binding the Numbers contained in this Volume 1s. 6d. Index to Vol. XXIX., 2½d., post free.**

**The Monthly Part for July is now ready. Price 6d., by post 8d.**

### SCRAPS AND GLEANINGS.

HER MAJESTY THE QUEEN has graciously approved of the new hospital steamer, now being built for work amongst the North Sea fishing fleets by the Royal National Mission to Deep Sea Fishermen, being called the *Queen Alexandra*. Her Majesty is the Society's Patron.

THE number of in-patients admitted during the past year to the East Suffolk and Ipswich Hospital was larger than in any previous year of its existence, being 1,140. The number of out-patients treated was 7,450, the total expenditure during the year was £6,026 odd, and the total receipts were £5,489 odd, the subscriptions showed an increase of £280, but more funds are still needed to enable the authorities to treat the increased number of patients. With a view of wiping off the adverse balance it was resolved at the recent annual meeting of the institution to transfer a sum of £2,000 from the capital to the income account.

THE Health Committee of the Sunderland Corporation have subscribed £150 for two beds in the sanatorium of the Society for the Prevention and Cure of Consumption in the County of Durham, and they have adopted tentatively for the year 1901 voluntary notification, paying half-a-crown for each case. The cost of providing this sanatorium was: For altering the existing buildings and furnishing for 12 patients, £699; for building new bedrooms and furnishing them for seven patients, £260; while the cost of building and furnishing the new wing will be £1,400. Towards the last two items the Committee have only available the sum of £840, and they urgently appeal for the balance of £820, as well as an additional £1,000 necessary to increase the accommodation to 40 beds.

### THE STUDENT OF MEDICINE.

### APPOINTMENTS.

**W. H. M. Alexander**, M.B., Ch.M.Glasg., appointed District Medical Officer of the Goole Union. **T. L. Anderson**, M.B.Melb., appointed Special Medical Officer for Plague at Fremantle, Western Australian. **Thos. Wm. N. Barlow**, M.R.C.S., L.R.C.P.Lond., D.P.H.Camb., appointed Medical Officer of Health for the Borough of Bootle, Visiting Medical Officer to the Linacre Hospital and Surgeon to the Police Force and Fire Brigade. **C. T. Bishop**, M.D.Lond., appointed Assistant Medical Officer at the Central London Sick Asylum District at Hendon. **G. H. S. Blackburne**, M.B., Ch.B.Melb., appointed Special Medical Officer for Plague at Perth, Western Australia. **W. F. Victor Bonney**, M.D., M.S.Lond., F.R.C.S., M.R.C.S., appointed Registrar to the Chelsea Hospital for Women. **Richard Hugh Brew**, L.R.C.P., appointed Medical Officer of Health for the Sutton Rural District. **G. C. R. Bull**, M.B.Lond., F.R.C.S.Eng., appointed District Medical Officer of the Newport Pagnell Union. **S. A. Coad**, M.R.C.S., L.R.C.P.Lond., appointed District Medical Officer of the Luton Union. **Ludford Cooper**, M.R.C.S., L.R.C.P.Lond., appointed Assistant Ophthalmic Surgeon to St. Bartholomew's Hospital, Rochester. **L. J. Crinion**, M.B., B.Ch., R.U.I., appointed Medical Officer for the Blessington No. 1 Dispensary District of the Naas Union. **A. C. Evered**, L.R.C.P., L.R.C.S.Edin., appointed Special Medical Officer for Plague at Perth, Western Australia. **P. J. Godfrey**, L.R.C.P.Edin., appointed Health Officer at Port Strahan, and also Medical Officer at Port Macquarie, Tasmania. **John Turner Grierson**, M.B., Ch.B.Vict., appointed Resident Medical Officer to the Liverpool Corporation Hospitals. **A. Gordon Gullan**, M.D.Lond., F.R.C.S.Eng., appointed Honorary Physician to the Stanley Hospital, Liverpool. **R. Halpin**, L.R.C.P.I., M.R.C.S.Eng., appointed Certifying Factory Surgeon for the Arklow District of the county of Wicklow. **G. C. Harper**, M.B., appointed House-Physician to the Perth Public Hospital. **Lewis D. Heather**, M.R.C.S., L.R.C.P.Lond., appointed Medical Officer of Health to the Hay and Bradwardine Rural District Council. **J. L. Henderson**, M.B., appointed Health Officer at North Ovens, Victoria. **A. Hendry**, M.B., Ch.B., appointed Resident Surgeon to the Southland Hospital, Invercargill, New Zealand. **Noel Lockwood Hood**, M.D.Camb., B.C., appointed an Honorary Surgical Officer to the York County Hospital. **F. C. W. Hounsell**, M.R.C.S.Eng., L.R.C.P.Lond., appointed District Medical Officer of the Weobley Union. **F. J. Ingoldby**, M.R.C.S.Eng., L.R.C.P.Edin., appointed Quarantine Officer at Albany, Western Australia. **O. Clayton**

**Jones**, M.B.Oxon., L.R.C.P.Lond., appointed Medical Officer and Public Vaccinator for the Ilfracombe District of the Barnstaple Union. **R. W. Leethbridge,** M.B., Ch.M., appointed Medical Superintendent of the Sunbury Lunatic Asylum, Victoria. **Thomas H. Littlejohn,** M.B., C.M.Edin., D.P.H., appointed Medical Officer of Health for the Metropolitan Borough of Hampstead. **John Hedley Marsh,** M.R.C.S., L.R.C.P.Lond., appointed Honorary Surgeon to the Macclesfield General Infirmary. **F. H. Maturin,** M.R.C.S.Eng., L.R.C.P.Lond., appointed District Medical Officer of the Lymington Union. **Eva McCall,** M.B., B.S.Glasg., appointed Assistant Resident Medical Officer to the Birkenhead Union Infirmary. **M. Becket McCarthy,** L.R.C.P., L.R.C.S.Edin., L.F.P.S.Glasg., appointed one of the Honorary Surgeons of the Masterton Hospital, New Zealand. **J. McKenzie,** M.A., M.B., Ch.B.Aberd., appointed House-Surgeon to the West London Hospital. **H. H. Meikle,** M.B., M.S.Glasg., appointed Medical Officer of Health to the Darlington Rural Council. **Samson G. Moore,** M.B., Ch.B.Vict., D.P.H.Eng., appointed Medical Officer of Health for the Borough of Huddersfield. **E. W. Morris,** M.R.C.S., appointed Assistant Health Officer at Port Adelaide, South Australia. **Herbert S. Pendlebury,** F.R.C.S., appointed Surgeon to the Royal Hospital for Children and Women, Waterloo Bridge Road. **A. W. Potts,** M.A.Cantab., M.D., C.M.Edin., appointed Assistant to the Professor of Therapeutics in the University of Birmingham. **Knowles Renshaw,** M.A., M.D., D.P.H.Cantab., appointed Assistant Medical Officer to the Manchester Hospital for Consumption and Diseases of the Throat and Chest. **J. C. R. Robinson,** M.R.C.S., L.R.C.P.Lond., appointed Certifying Factory Surgeon for the Harleston District of Norfolk. **J. C. Saunders,** L.R.C.P.Edin., M.R.C.S.Eng., appointed Medical Officer for the Bootle Division of the West Derby Union. **A. Marmaduke Sheild,** F.R.C.S., appointed Consulting Surgeon to the Royal Hospital for Children and Women, Waterloo Bridge Road. **A. H. Shepard,** M.B., B.S.Dub., appointed District Medical Officer of the Macclesfield Union. **J. W. Smith,** M.B., C.M.Edin., F.R.C.S.Eng., appointed Honorary Assistant Surgeon to the Manchester Royal Infirmary. **W. Spencer,** L.R.C.P., L.R.C.S.Edin., appointed Medical Officer of the Bolsover District of the Chesterfield Union. **W. S. V. Stock,** M.B.Lond., M.R.C.S.Eng., appointed Resident Medical Officer to the Manchester Royal Infirmary. **P. M. Stoms,** M.R.C.S.Eng., L.S.A., appointed District Medical Officer of the East Ham Union. **C. E. Tanner,** M.D.Durh., F.R.C.S.Eng., appointed Medical Officer of Health for the Farnham Rural District. **Thos. E. Frazer Toovey,** L.R.C.P., L.R.C.S.Edin., L.F.P.S.Glasg., appointed Assistant Resident Medical Officer to St. Mary's Hospital for Sick Children, Plaistow. **A. C. K. Turner,** M.B., C.M.Edin., appointed Medical Officer for the Third District of the Northleach Union. **B. R. Turner,** M.B.Camb., appointed District Medical Officer of the Oundle Union. **F. J. Waldo,** M.D.Camb., M.R.C.S., D.P.H.Eng., appointed Coroner for the City of London. **Arthur E. Whitehead,** L.R.C.P., L.R.C.S.Edin., L.F.P.S.Glasg., appointed Resident Medical Officer to St. Mary's Hospital for Sick Children, Plaistow. **Herbert Williams,** M.D.Lond., D.P.H.Camb., appointed Medical Officer of Health for the Port of London. **E. C. Wimberley,** L.S.A., appointed District Medical Officer of the Towcester Union. **Oliver Withers,** M.D.Durh., M.R.C.S.Eng., appointed Medical Officer of Health to the Sale District Council.

